# The Resuscitation of Apparently Stillborn Neonates: A Peek Into the Practice in China

**DOI:** 10.3389/fped.2020.00231

**Published:** 2020-06-02

**Authors:** Xueyu Chen, Huitao Li, Jingyu Song, Panpan Sun, Binchun Lin, Jie Zhao, Chuanzhong Yang

**Affiliations:** Department of Neonatology, Affiliated Shenzhen Maternal and Child Healthcare Hospital, Southern Medical University, Shenzhen, China

**Keywords:** apparent stillbirth, asphyxia, resuscitation, outcome, China

## Abstract

Apparently stillborn neonates are born in the terminal stage of secondary apnoea and respond poorly to basic resuscitation procedures proposed by the Neonatal Resuscitation Program (NRP). Increasing experimental and clinical evidence shows that stringently adhering to the NRP guidelines may delay sufficient ventilation and chest compressions and consequently prolong the duration of asystole in apparently stillborn neonates. To add to this information, we summarized our experience with the resuscitation of apparently stillborn neonates and reported the neonatal outcomes in a cohort of apparently stillborn neonates resuscitated at a tertiary care center in China.

## Introduction

Although the definition of apparent stillbirth is inconclusive, it generally refers to a severely asphyxiated neonate with a 1-min Apgar score (AS1) of 0–1 in a clinical setting (1–3), particularly a neonate who had a heartbeat before or during labor. It is estimated that 44% of stillbirths and 73% of newborn deaths occur around the time of labor and within the first week after birth, with birth asphyxia among the most common causes of deaths (4). In 2014, the Every Newborn Action Plan (ENAP) was endorsed by the World Health Assembly. It was developed to reduce newborn deaths and stillbirths (5) and highlights the apparently stillborn population whose deaths are likely to be prevented with rapid and effective resuscitation.

Apparently stillborn neonates are often born in the stage of secondary apnoea, a situation in which their central nervous system and direct diaphragmatic functioning are depressed due to prolonged hypoxia (6). Secondary apnoea may last for 5–8 min before death occurs (7). In addition, experimental evidence shows that the myocardium in neonates is more vulnerable to ischemia than the myocardia in juveniles and adults (8). Furthermore, the outcomes (including survival and cerebral disabilities) of resuscitation mostly depend on the duration of asystole. Therefore, resuscitation in apparently stillborn neonates aims to shorten the duration of asystole.

Although the guidelines on the resuscitation of neonates jointly proposed by the American Academy of Pediatrics and American Heart Association (AAP/AHA) are internationally recognized, the guideline may not be very applicable in this unique population of apparent stillbirths. Recently, Sharma et al. investigated the time that resuscitators needed to perform the steps required for corrective ventilation (MRSOPA) and the time to initiate chest compressions in bradycardic and asystolic newborns. The authors reported that many resuscitators preferred early endotracheal intubation instead of the MRSOPA method considering that corrective steps may delay the initiation of intubation and chest compressions in newborns with severe cardiovascular compromise (9). In our experience, the resuscitation procedures according to the NRP guidelines might postpone the recovery of apparently stillborn neonates.

**Figure 1:**
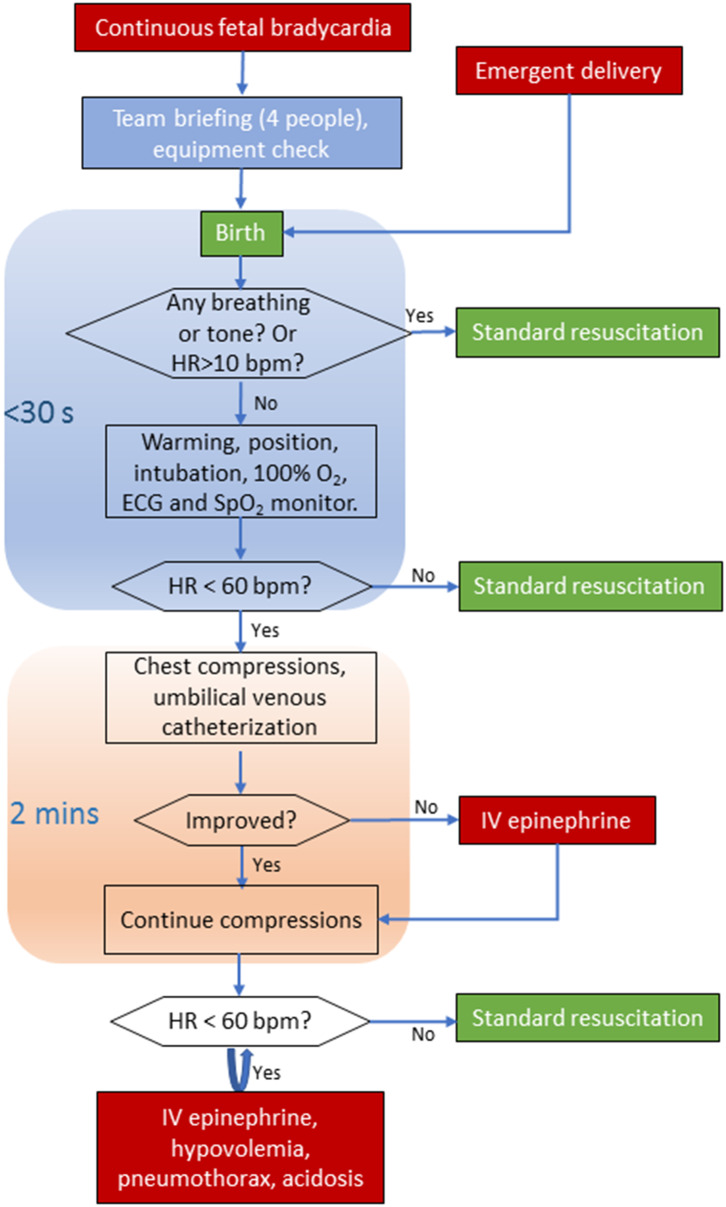
Algorithm for the resuscitation of asphyxiated newborns with asystole or severe bradycardia. ECG, electrocardiography; SpO_2_, saturation pulse oxygen; HR, heart rate; bpm, beats per minute; IV, intravenous.

In this report, our institutional resuscitation practice for apparent stillborns is systematically described (Figure 1) and compared with the NRP (10) and Chinese neonatal resuscitation guidelines [(11), Table 1].

**Table 1:** Comparison of apparently stillborn neonate resuscitation algorithms with international guidelines (NRP, 2015) and Chinese neonatal resuscitation guidelines (CNR, 2016).

	**NRP algorithm**	**CNR algorithm**	**Apparent stillbirth algorithm**	**Illustration**
Team	Three people	Three people	Four people	(1) Resuscitation of apparently stillborn neonates often requires intubation, heart compressions, and umbilical venous catheter (UVC) placement simultaneously; therefore, multiple people are needed.
Assessment	Term, tone, breathing, or crying	Term, clean amniotic fluid, tone, breathing, or crying	Any breathing, tone or HR > 10 bpm?	(1) Apparently stillborn neonates are mostly in asystolia or approaching it (HR < 10 bpm). (2) Infants may die from complications of MAS [such as pneumothorax and persistent pulmonary hypertension (PPHN)] in low-resource areas of China. Therefore, to prevent subsequent complications, the CNR still recommends suctioning the airway in fluid-contaminated newborns with low vitality^*^.
Drying and stimulation	Yes	Yes	No	Apparently stillborn neonates are at the terminal stage of secondary apnoea, and stimulation does not help (13). In addition, apparently stillborn infants often need therapeutic hypothermia later to prevent brain injury; warming and drying are less important than aeration in this case.
Intubation	Initiate after 1 min (if needed)	Initiate after 1 min (if needed)	Preferably finish within 30 s	(1) Intubation facilitates the recovery of heart beat and cerebral perfusion. (2) Heart compression is mostly needed in resuscitation of apparent stillbirth and NRP guidelines recommend to intubate before heart compression. (3) Only experienced neonatologists are allowed to perform the resuscitation of apparent stillbirths in most level III centers in China, which ensures intubation to be accomplished within 20 s and less intubation-related injury.
Initiate FiO_2_%	>35 weeks 21%, <35 weeks 21–30%	21, 40, and 100%^**^	100%	Evidences about the injury of 100% oxygen are slightly conflicting. According to our experience, restoring cerebral perfusion is the top priority in these infants and recommend using pure oxygen to initiate ventilation; however, we are always aware of potential hyperoxia injury.
Frequency of ventilation	40–60 times/min	40–60 times/min	>60 times/min	Facilitate aeration and restore oxygenation in cerebral blood.
First assessment of heart rate	100 bpm	100 bpm	10 bpm	Apparently stillborn neonates are mostly in asystolia or approaching to it.
Initiation of chest compressions	After 1–2 min if needed	After 1–2 min if needed	After 30 s	Timely chest compressions facilitate perfusion of well-oxygenated blood throughout the body, preventing organ injury caused by hypoxia.
Frequency of heart compression	90 times/min	90 times/min	>120 times/min	Facilitate perfusion of the well-oxygenated blood throughout the body.
Chest compression to ventilation ratio	3:1	3:1	Not restricted to 3:1	Evidence from piglets shows sustained inflations (SIs) combined with chest compressions significantly shortened the time needed to induce spontaneous circulation compared to the standard 3:1 compression: ventilation ratio (14).
UVC	After ~2 min	After ~2 min	After 30 s, initiate at the same time as chest compressions	Epinephrine is often needed in the resuscitation of apparently stillborn neonates. Therefore, the UVC can be placed concurrently with chest compressions.
First dose of epinephrine	After ~4 min	After ~4 min	After 1.5 min, if needed.	Since the UVC is established early, epinephrine can be administered early if needed.

*The key to resuscitation of apparently stillborn neonates is to deliver oxygenated blood to the body. Therefore, never start chest compressions before the establishment of effective ventilation*.

* *Low vitality refers to one of the following symptoms: low tone, no breathing or gasping, HR < 100 bpm*.

** *In rural areas of China, self-inflating bags are the most common devices for resuscitation. Air-oxygen mixers and blenders are not available. The CNR is aware of the toxicity of 100% oxygen and therefore suggests removing the corrugated tube from the self-inflating bag to generate a concentration of 40% oxygen after mixing ambient air with 100% oxygen*.

## Definition of Apparent Stillbirth in this Report

As mentioned above, there is no consensus on the definition of apparent stillbirth worldwide. Apparent stillbirth in this report refers to neonates with continuous fetal bradycardia (<60 bpm), born with apnea, no muscle tone, breath or reflex, and pale in color immediately after birth. When heart auscultation is performed, it is usually when a heartbeat of around 0–1 beats within 6 s (which equates to a heart rate of 0–10 bpm) is detected. For these neonates, any delay in initiating effective resuscitation will lead to a 4-fold longer period to restore respiration (12, 13).

## Key Points in our Resuscitation of Apparently Stillborn Neonates

1. **Team assembly**. Since intubation, chest compression, and umbilical venous catheterization may need to be performed concurrently, an experienced team made up of at least four skilled caregivers is required.
2. **Ventilation**. Intubate the baby immediately and start ventilation with an oxygen concentration of 100% if breath and muscle tone are both absent, and HR is below 10 bpm (0–1 beats within 6 s). Concomitantly, electrocardiography (ECG, 3-lead) and pulse oxygen monitors should be connected to facilitate heart rate (HR) assessment. ECG enables resuscitators to obtain an HR without interrupting chest compressions. These procedures should be completed within 30 s. The flow of oxygen can be increased to 10–15 L/min to ensure the delivery of oxygen. An initial peak inspiratory pressure (PIP) of 30 cmH_2_O is recommended. In the case of tracheal obstruction (such as meconium-stained amniotic fluid, MAS), endotracheal suction can be performed to facilitate ventilation. We recommend a PIP of 30 cmH_2_O at the initiation of ventilation and modifications should be monitored closely.
3. **Chest compressions**. Chest compressions should be started immediately if the HR is below 60 bpm after intubation is performed. In apparently stillborn neonate resuscitation, the frequencies of both chest compressions and ventilation are increased and the ratio is not restricted to 3:1 (14). A ratio between 2:1 and 3:1 is recommended to facilitate aeration in the resuscitation of apparent stillbirth. Chest compressions should be performed 120–140 times/min (resembling the average HR of a newborn), and ventilation should be performed 60–80 times/min. Generally, compression and ventilation are still synchronized, but it can also be unsynchronised if the infant's condition does not improve. If the baby's HR improves to above 60 bpm, a return to standard resuscitation is considered. If the HR does not improve after 1 min of chest compressions, immediate intravascular administration of epinephrine is recommended. If the HR improves but remains under 60 bpm, epinephrine can be given after 2 min of continuous chest compressions.

## Outcomes of Apparently Stillborn Neonates Resuscitated in the Local Ward

The Shenzhen Maternal and Child Healthcare Hospital (SMCHH) is a tertiary center with an average of 15,000 births per year. From 2003 to 2018, there were 232,043 registered births at SMCHH. During the study period, 50 newborns termed apparently stillborn (gestation: 39 ± 2 weeks; weight: 2,978 ± 701 g) were successfully resuscitated and transferred to the neonatal intensive care unit (NICU). A total of 38 (76%) survived until discharge, among whom 50% (19/38) had no obvious morbidity at discharge. A total of 31.6% (12/38) of survivors were diagnosed with moderate or severe hypoxic-ischemic encephalopathy (HIE).

## Discussion

Apparently stillborn neonates are mostly born at the terminal stage of secondary apnea (15). If they are left untreated or not resuscitated timely and sufficiently, death often occurs (13). Apparently stillborn neonates are different from neonates born with primary apnea, who generally have noticeable tone or reflex and respond to drying and stimulation. A very small proportion of neonates at primary apnea might also show no vital signs, they are difficult to be differentiated from apparent stillbirth. Nevertheless, no vital sign itself also indicates a severe condition approaching the secondary apnoea. Furthermore, neonates in the primary apnea hardly have an HR below 10 bpm and are not applied with this algorithm.

Resuscitation for apparently stillborn neonates aims to deliver the well-oxygenated blood to the hypoxic-ischaemic organs in a time as short as possible to avoid cerebral damage in these infants. Therefore, ventilation is considered the top priority. Drying, suction, and stimulation will not work in neonates with secondary apnoea (13). Experimental evidence has demonstrated that even adequate positive pressure ventilation (PPV) failed to increase the HR within a short time in bradycardic newborn piglets (16). Therefore, we recommend that apparently stillborn newborns are intubated immediately after birth for an effective and speedy recovery of the heart beat. Cardiac compression is often needed in the resuscitation of asystolic neonates which requires intubation to improve ventilation efficacy (Textbook of neonatal resuscitation 7th edition, page 118) as recommended by NRP guidelines. Although no studies clearly demonstrate that restoration of heart rate is faster with intubation than with mask ventilation, at least 30% air leak during mask PPV were found in the delivery rooms (17). In contrast to many centers in other countries, only experienced neonatologists are allowed to perform the resuscitation of apparent stillbirths in most level III centers in China, which ensures intubation to be accomplished within 20 s and less intubation-related injury. This recommendation is also supported by Berglund et al., who recommended direct intubation in neonates with asystolia (18). Besides, to reduce “excessive resuscitation” we suggest returning to the standard resuscitation if neonates' performance improves after each assessment. There might still be some neonates who would be excessively intubated, however, the risk of delayed ventilation in neonates with secondary apnea outweighs the risk of over resuscitation in neonates with primary apnea given that differentiation between primary and secondary apnea is challenging in this population.

Investigating the concentration of oxygen used for initial resuscitation has long been a focus of interest. Hyperoxia may increase the risk of brain injury due to loss of function in overvasodilated cerebral vessels (19). In contrast, evidence from severely hypoxic-ischaemic rats suggested that no difference was observed in the rate of brain injury between 100 and 21% oxygen (20). Solevåg et al. recently performed a literature review on whether to use 21 or 100% oxygen in the resuscitation of severely asphyxiated neonates and concluded that resuscitation with 100% oxygen may restore cerebral blood flow more rapidly than resuscitation with 21% oxygen but cautioned that pure oxygen may damage the ischaemic brain (21). The brains of apparently stillborn newborns remain under hypoxic-ischaemic conditions for a relatively long period, and there is a balance between prolonging this period and causing hyperoxia-related injury. According to our experience, restoring cerebral perfusion is the top priority in these infants therefore we recommend using pure oxygen to initiate ventilation; however, we are always aware of potential hyperoxia injury.

The pressure during PPV is also different between intensive resuscitation and standard guidelines. Since an adequate PIP is instrumental for the swift establishment of ventilation in emergencies, we recommend starting with a PIP of 30 cmH_2_O and modifying it according to the experience of the resuscitators. Notably, it is crucial to lower the PPV promptly to avoid pneumothorax, overventilation, and hypocarbia, which are also associated with brain injury (22).

Studies on the outcomes of stillbirths are difficult and limited. Our hospital's survival rate at discharge is 76%, which is higher than the 62% which was reported in 42 successfully resuscitated apparently stillborn neonates during the period 1986–1994 by Casalaz et al. (23). Nelson et al. reported a survival rate of 83.9% at discharge in 93 successfully resuscitated apparently stillborn neonates during 2002–2007 (2); this rate was slightly higher than that in our center. This may be because parental responsibility for their child's medical costs has increased, and parents receive little social help in China. In our center, most of the neonatal deaths were due to the withdrawal of intensive care as decided by the parents.

We summarized neonatal morbidities in apparently stillborn infants in the NICU who survived. Nelson et al. reported a similar rate of 33.7% (28/83) for significant neonatal morbidities, including seizure and HIE (2). The long-term outcomes of survivors were not reported in their study. Casalaz et al. reported that 62% of neonate survivors (16/26) were free of morbidities at 20 months and at 8 years later (23). Notably, these studies were performed decades ago. There is an urgent need to assess the long-term outcomes of apparently stillborn infants.

Unfortunately, we do not have complete data on the number of apparent stillbirths during the study period. This algorithm is mostly based on clinical experience. While experience is not evidence, in the absence of evidence, it is likely to be the best option for guiding clinical practice. Nevertheless, it is important to perform a trial or quality assurance study to evaluate the effectiveness of the algorithm in these severely asphyxiated neonates although the logistic and ethical principles are challenging if not impossible to overcome. Additionally, resuscitation of apparent stillbirth significantly relies on experienced neonatologists with skilled intubation and venous catheterization, which might reduce the transferability of our algorithm to the units without adequate healthcare settings.

The ILCOR guidelines are based on currently available evidence through a vigorous and consensus-driven process. The guidelines are widely recognized and appropriate for the resuscitation of neonates under most circumstances. However, how to resuscitate apparent stillbirth was not stated in the guidelines. Lack of evidence suggests that ILCOR guidelines are applicable to this unique population. Therefore, our algorithm proposed in this report is not challenging the ILCOR guidelines, but an adjunctive specific to apparent stillbirths.

In conclusion, accumulating experimental evidence and clinical practice experience suggest that standard resuscitation guidelines may not precisely apply to apparently stillborn neonates and to restore oxygenation and perfusion in a timely manner, we propose to initiate intubation and chest compressions immediately in apparently stillborn neonates and summarize our institutional algorithm.

## Data Availability Statement

The raw data supporting the conclusions of this article will be made available by the authors, without undue reservation, to any qualified researcher.

## Ethics Statement

The Shenzhen Maternity and Child Health Care Hospital Institutional Ethical Committee approved the collection and usage of the clinical information for research purposes and waived the requirement for informed consent (IEC No. [2019]-119).

## Author Contributions

CY conceptualized and designed the study. XC and HL constructed the flowchart. PS and JS carried out the clinical data collection and data analysis. XC wrote the first draft of this manuscript. BL, JZ, and CY reviewed and revised the manuscript. All authors read and approved the final manuscript.

## Conflict of Interest

The authors declare that the research was conducted in the absence of any commercial or financial relationships that could be construed as a potential conflict of interest.

## References

[1] Manley BJ Owen LS Hooper SB Jacobs SE Cheong JLY Doyle LW. Towards evidence-based resuscitation of the newborn infant. Lancet. (2017) 389:1639–48. 10.1016/S0140-6736(17)30547-0 28443558

[2] Nelson K Simonsen SE Henry E Wilder S Rose NC. The apparently stillborn infant: risk factors, incidence, and neonatal outcome. Am J Perinatol. (2011) 28:75–82. 10.1055/s-0030-1262906 20645239

[3] La Gamma EF Lena Kim J Shah S. Resuscitation of potentially stillborn periviable neonates: who lives, who dies and who gets missed? Acta Paediatr. (2016) 105:1252–4. 10.1111/apa.13591 27726188

[4] Oza S Cousens SN Lawn JE. Estimation of daily risk of neonatal death, including the day of birth, in 186 countries in 2013: a vital-registration and modelling-based study. Lancet Global Health. (2014) 2:e635–44. 10.1016/S2214-109X(14)70309-2 25442688

[5] Kinney MV Cocoman O Dickson KE Daelmans B Zaka N Rhoda NR. Implementation of the every newborn action plan: progress and lessons learned. Semin Perinatol. (2015) 39:326–37. 10.1053/j.semperi.2015.06.004 26249104

[6] Groenendaal F de Vries LS. Fifty years of brain imaging in neonatal encephalopathy following perinatal asphyxia. Pediatric Res. (2017) 81:150–5. 10.1038/pr.2016.195 27673422

[7] Kattwinkel J Textbook of Neonatal Resuscitation. Dallas, TX: American Heart Association (2000).

[8] Parrish MD Payne A Fixler DE. Global myocardial ischemia in the newborn, juvenile, and adult isolated isovolumic rabbit heart. Age-related differences in systolic function, diastolic stiffness, coronary resistance, myocardial oxygen consumption, extracellular pH. Circ Res. (1987) 61:609–15. 10.1161/01.RES.61.5.609 3664973

[9] Sharma V Lakshminrusimha S Carrion V Mathew B. Resuscitator's perceptions and time for corrective ventilation steps during neonatal resuscitation. Resuscitation. (2015) 91:63–6. 10.1016/j.resuscitation.2015.03.008 25796996 PMC4803469

[10] Wyckoff MH Aziz K Escobedo MB Kapadia VS Kattwinkel J Perlman JM. Part 13: Neonatal resuscitation: 2015 american heart association guidelines update for cardiopulmonary resuscitation and emergency cardiovascular care (reprint). Pediatrics. (2015) 136:S196–218. 10.1542/peds.2015-3373G 26471383

[11] Chinese neonatal resuscitation committee China neonatal resuscitation guideline 2016. Chin J Perinat Med. (2016) 19:481–6. 10.3760/cma.j.issn.1007-9408.2016.07.001

[12] Dawes GS Jacobson HN Mott JC Shelley HJ Stafford A. The treatment of asphyxiated, mature foetal lambs and rhesus monkeys with intravenous glucose and sodium carbonate. J Physiol. (1963) 169:167–84. 10.1113/jphysiol.1963.sp007248 14078056 PMC1368709

[13] Moshiro R Mdoe P Perlman JM. A global view of neonatal asphyxia and resuscitation. Front Pediatr. (2019) 7:489. 10.3389/fped.2019.00489 31850287 PMC6902004

[14] Li ES Gorens I Cheung PY Lee TF Lu M O'Reilly M Chest compressions during sustained inflations improve recovery when compared to a 3:1 compression: ventilation ratio during cardiopulmonary resuscitation in a neonatal porcine model of asphyxia. Neonatology. (2017) 112:337–46. 10.1159/000477998 28768280

[15] Wylie J Bruinenberg J Lacovidou N Tinnion R Kreth U Hampshire S Newborn Life Support. Niel: European Resuscitation Council VZW (2015).

[16] Espinoza ML Cheung PY Lee TF O'Reilly M Schmolzer GM Heart rate changes during positive pressure ventilation after asphyxia-induced bradycardia in a porcine model of neonatal resuscitation. Arch Dis Child Fetal Neonatal Ed. (2019) 104:F98–101. 10.1136/archdischild-2017-314637 29778994

[17] Schmölzer GM Morley CJ Kamlin O. Enhanced monitoring during neonatal resuscitation. Semin Perinatol. (2019) 43:151177. 10.1053/j.semperi.2019.08.006 31493856

[18] Berglund S Norman M Grunewald C Pettersson H Cnattingius S. Neonatal resuscitation after severe asphyxia–a critical evaluation of 177 Swedish cases. Acta Paediatr. (2008) 97:714–9. 10.1111/j.1651-2227.2008.00803.x 18460105 PMC2430333

[19] Sobotka KS Ong T Polglase GR Crossley KJ Moss TJ Hooper SB. The effect of oxygen content during an initial sustained inflation on heart rate in asphyxiated near-term lambs. Arch Dis Child Fetal Neonatal Ed. (2015) 100:F337–43. 10.1136/archdischild-2014-307319 25922189

[20] Smit E Liu X Gill H Jary S Wood T Thoresen M. The effect of resuscitation in 100% oxygen on brain injury in a newborn rat model of severe hypoxic-ischaemic encephalopathy. Resuscitation. (2015) 96:214–9. 10.1016/j.resuscitation.2015.07.050 26300234

[21] Solevag AL Schmolzer GM Cheung PY Is supplemental oxygen needed in cardiac compression?-the influence of oxygen on cerebral perfusion in severely asphyxiated neonates with bradycardia or cardiac asystole. Front Pediatr. (2019) 7:486 10.3389/fped.2019.00486 31824899 PMC6879425

[22] Pappas A Shankaran S Laptook AR Langer JC Bara R Ehrenkranz RA. Hypocarbia and adverse outcome in neonatal hypoxic-ischemic encephalopathy. J Pediatr. (2011) 158:752–8.e1. 10.1016/j.jpeds.2010.10.019 21146184 PMC3229432

[23] Casalaz DM Marlow N Speidel BD. Outcome of resuscitation following unexpected apparent stillbirth. Arch Dis Child Fetal Neonatal Ed. (1998) 78:F112–5. 10.1136/fn.78.2.F112 9577280 PMC1720775

